# Is Circumferential Minimally Invasive Surgery Effective in the Treatment of Moderate Adult Idiopathic Scoliosis?

**DOI:** 10.1007/s11999-014-3565-2

**Published:** 2014-03-22

**Authors:** Neel Anand, Eli M. Baron, Babak Khandehroo

**Affiliations:** 1 Spine Center, Cedars-Sinai Medical Center, 444 S San Vicente Blvd, Suite 800, Los Angeles, CA 90048 USA; 2 Department of Neurosurgery, Cedars Sinai Medical Center, Los Angeles, CA USA

## Abstract

**Background:**

Outcomes for minimally invasive scoliosis correction surgery have been reported for mild adult scoliosis. Larger curves historically have been treated with open surgical procedures including facet resections or posterior column osteotomies, which have been associated with high-volume blood loss. Further, minimally invasive techniques have been largely reported in the setting of degenerative scoliosis.

**Questions/purposes:**

We describe the effects of circumferential minimally invasive surgery (cMIS) for moderate to severe scoliosis in terms of (1) operative time and blood loss, (2) overall health and disease-specific patient-reported outcomes, (3) deformity correction and fusion rate, and (4) frequency and types of complications.

**Methods:**

Between January 2007 and January 2012, we performed 50 cMIS adult idiopathic scoliosis corrections in patients with a Cobb angle of greater than 30° but less than 75° who did not have prior thoracolumbar fusion surgery; this series represented all patients we treated surgically during that time meeting those indications. Our general indications for this approach during that period were increasing back pain unresponsive to nonoperative therapy with cosmetic and radiographic worsening of curves. Surgical times and estimated blood loss were recorded. Functional clinical outcomes including VAS pain score, Oswestry Disability Index (ODI), and SF-36 were recorded preoperatively and postoperatively. Patients’ deformity correction was assessed on pre- and postoperative 36-inch (91-cm) standing films and fusion was assessed on CT scan. Minimum followup was 24 months (mean, 48 months; range, 24–77 months).

**Results:**

Mean blood loss was 613 mL for one-stage surgery and 763 mL for two-stage surgery. Mean operative time was 351 minutes for one-stage surgery and 482 minutes for two-stage surgery. At last followup, mean VAS and ODI scores decreased from 5.7 and 44 preoperatively to 2.9 and 22 (p < 0.001 and 0.03, respectively) and mean SF-36 score increased from 48 preoperatively to 74 (p = 0.026). Mean Cobb angle and sagittal vertical axis decreased from 42° and 51 mm preoperatively to 16° and 27 mm postoperatively (both p < 0.001). An 88% fusion rate was confirmed on CT scan. Perioperative complications occurred in 11 of the 50 patients (22%), with delayed complications needing further surgery in 10 more patients at last followup.

**Conclusions:**

cMIS provides for good clinical and radiographic outcomes for moderate (30°–75°) adult idiopathic scoliosis. Patients undergoing cMIS should be carefully selected to avoid fixed, rigid deformities and a preoperative sagittal vertical axis of greater than 10 cm; surgeons should consider alternative techniques in those patients.

**Level of Evidence:**

Level IV, therapeutic study. See Instructions for Authors for a complete description of levels of evidence.

## Introduction

In recent years, advances in technology have allowed many spinal conditions to be treated in a less invasive fashion. These techniques allow the surgeon to move away from open approaches involving extensive soft tissue destruction toward minimally invasive approaches resulting in less tissue trauma while performing a corrective procedure on the spine [12]. By limiting collateral surgical damage, minimally invasive spine procedures may result in decreased blood loss and pain and quicker return to daily activities [2, 5, 12, 19].

**Fig. 1A–E:**
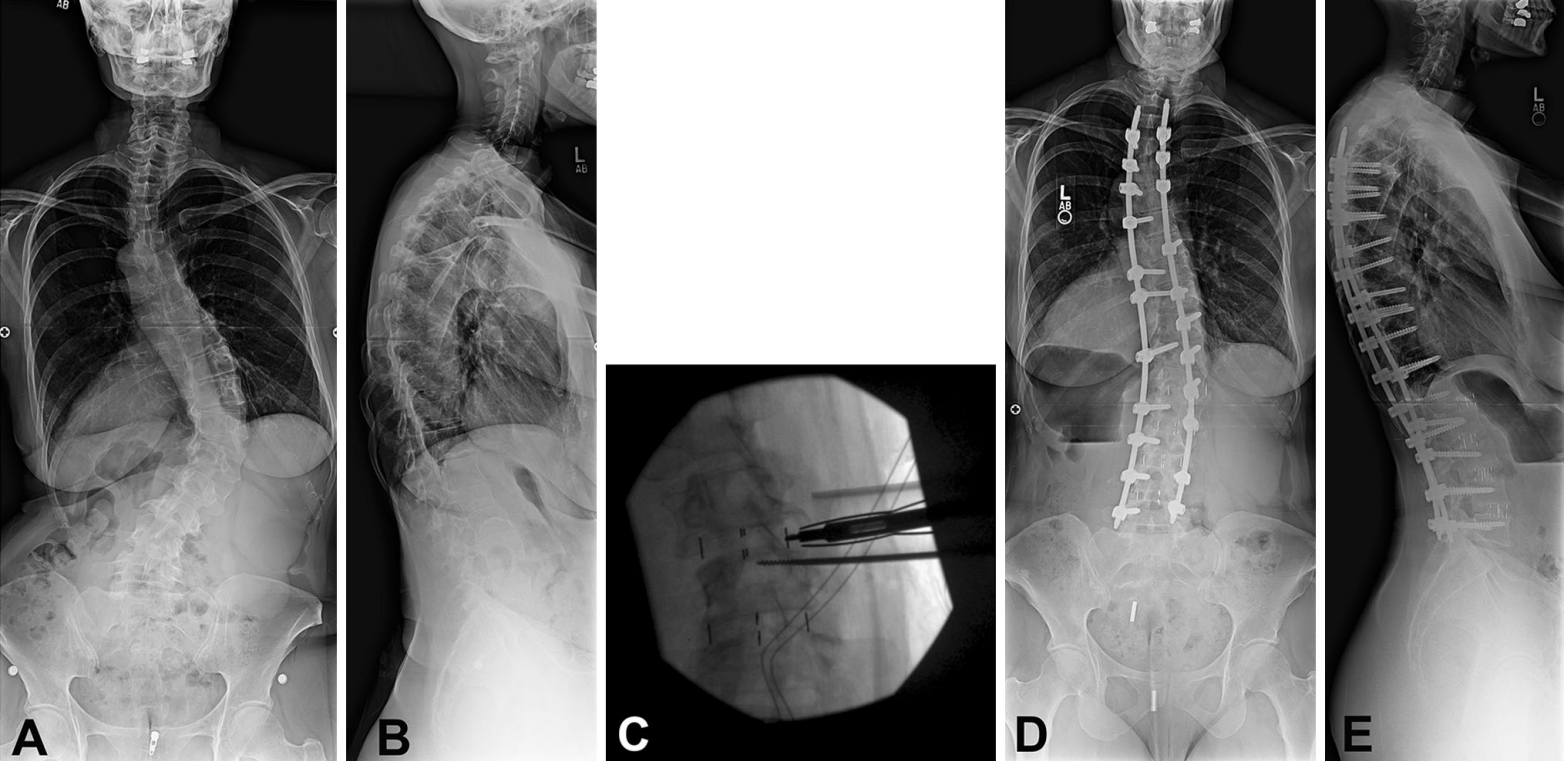
(**A**) AP and (**B**) lateral 36-inch standing films in a 65-year-old woman show a right thoracolumbar curve measuring approximately 55° from T8 to L3. She complained of back pain over her curve that was refractory to analgesics and nonoperative measures. (**C**) An intraoperative AP fluoroscopic image shows insertion of DLIF graft after transpsoas discectomy. (**D**) AP and (**E**) lateral 36-inch films taken 2 years after surgery show correction of her curve to approximately 27°. Sagittal balance is maintained.

When compared with open scoliosis correction, circumferential minimally invasive surgery (cMIS) has been shown to achieve comparable deformity correction in both the sagittal and coronal planes in mild to moderate cases of thoracolumbar scoliosis [4, 8, 16, 25]. Nevertheless, the majority of patients in published series are patients with degenerative scoliosis. Typically these patients present after the age of 40 years and without a history of adolescent scoliosis [22]. In contrast, adult idiopathic scoliosis (AIS) is a scoliotic deformity in patients older than 18 years and not typically developing de novo (Fig. 1A−B). This occurs in approximately 2% to 4% of adults younger than 45 years and its prevalence probably remains constant [9]. Adults with untreated or previously braced adolescent idiopathic scoliosis typically present with pain related to their curve and occasionally increasing radiographic and cosmetic spinal deformity. If nonoperative techniques fail to adequately treat their symptoms, surgery may be indicated [9, 24].

We therefore described the role of cMIS for AIS with regard to (1) operative time and blood loss, (2) overall health and disease-specific patient-reported outcomes, (3) the magnitude of deformity correction and fusion rates with this approach without osteotomies, and (4) the frequency and types of complications observed with this approach.

Of note, the first 11 patients included in this report were also reported on in a previous paper in *Spine* and some in *Clinical Orthopaedics and Related Research* ^®^ [3, 4]. The present paper specifically addresses and documents the results of cMIS techniques for the treatment of AIS and excludes patients with a diagnosis of de novo adult degenerative scoliosis. The work in *Spine* focused largely on patients with degenerative scoliosis and the earlier work in *Clinical Orthopaedics and Related Research* ^®^ focused on the utility of axial lumbar interbody fusion for L5-S1 fusion in scoliosis. This paper also has longer followup on those same 11 patients. The use of cMIS here is also of interest as conclusions regarding radiographic and overall health and disease-specific patient-reported outcomes in the setting of degenerative scoliosis may not be applicable to AIS.

## Patients and Methods

Data for this study were obtained through retrospective chart review with internal review board approval. We reviewed a database of 176 patients who underwent cMIS correction for adult scoliosis performed by the senior spine surgeon (NA) at a single tertiary academic center between January 2007 and January 2012. Seventy-six of these patients had true AIS with known scoliosis in adolescence and Cobb angles of greater than 30°. Fifty of these patients had a Cobb angle of less than 75°, had not had prior thoracolumbar fusion surgery, and were followed for a minimum of 24 months; this series represented all of the patients we treated surgically during that time meeting those indications. Our general indications for this approach during that period were increasing back pain unresponsive to nonoperative therapy with cosmetic and radiographic worsening of curves.

There were 13 men and 37 women, with a mean age of 61 years (range, 20–85 years) (Table 1). Forty-four patients had their apex at the lumbar or thoracolumbar level and six at the thoracic level. Thirty-six patients had preoperative radicular symptoms with stenosis on imaging studies. All underwent deformity correction and fusion using all or a combination of different cMIS strategies: direct lateral transpsoas interbody fusion (DLIF) (n = 44) (Fig. 1C) and L5-S1 axial lumbar interbody fusion (n = 28), followed by multilevel percutaneous pedicle screw fixation with free-hand rod placement (posterior instrumentation) (n = 50). Thirty-seven patients were staged with DLIF done first followed by the posterior instrumentation including axial lumbar interbody fusion done 3 days later. L5-S1 was included in the fusion whenever there were any degenerative changes, obliquity, fractional curve, stenosis, spondylolisthesis, or sagittal imbalance. None of our patients underwent any kind of posterior column osteotomy or facet resection. All patients had participated in extensive nonoperative therapies without adequate relief of their symptoms before being considered for surgery. None of the patients had prior fusion or fused facets on preoperative CT scanning. Thus, flexible and stiff curves were considered for surgery while truly rigid curves were excluded [22]. The mean number of levels operated on was seven (range, four to 15). The mean followup was 48 months (range, 24–77 months).

**Table 1:** Patient demographic data

Variable	Value
Number of patients	50
Male:female (number of patients)	13:37
Age (years)*	61 (20–85)
Number of segments operated on*	7 (4–15)

* Values are expressed as mean, with range in parentheses.

In all patients, recombinant human BMP-2 absorbable collagen sponges (Infuse^®^; Medtronic Sofamor Danek, Memphis, TN, USA) and Grafton^®^ putty demineralized bone matrix (Osteotech, Eatontown, NJ, USA) were used. Details of surgical techniques and recombinant human BMP-2 dosing have been described in our prior publications [3–5, 8].

Postoperative visits were scheduled at 6 weeks, 3 months, 6 months, 1 year, 2 years, and yearly thereafter. Clinical outcome data including VAS, Oswestry Disability Index (ODI), and SF-36 were prospectively collected at each visit through self-administered patient questionnaires. Standing deformity 36-inch (91-cm) films were taken at all postoperative visits (Fig. 1D−E). Cobb angles, sagittal balance (sagittal vertical axis), coronal balance, lumbar apical vertebral translation, and pelvic incidence-lumbar lordosis mismatch were measured. Additionally, CT scanning was performed at minimum 1 year postoperatively, where the presence of bridging bone in and around interbody grafts was looked for, in addition to fused facets on sagittal and coronal reconstructions with lack of any lucencies around screws and grafts [4]. Fusion assessment was performed by a research associate (BK) experienced in analyzing radiographs and CT scans.

Unpaired t-tests were used to calculate significance of postoperative clinical outcomes and radiographic measurements; all calculations were performed using Microsoft^®^ Excel^®^ (Microsoft Corp, Redmond, WA, USA).

## Results

For patients with one-stage same-day surgery, the mean blood loss was 613 mL (range, 150–1500 mL) and the mean surgical time was 351 minutes (range, 176–510 minutes) (Table 2). Patients with two-stage surgery had a mean blood loss of 763 mL (range, 25–2500 mL), with 327 mL (range, 25–2100 mL) for the first stage and 463 mL (range, 100–2500 mL) for the second stage. The mean surgical time was 482 minutes (range, 83–546 minutes), with 192 minutes (range, 83–531 minutes) for the first stage and 291 minutes (range, 153–546 minutes) for the second stage.

**Table 2:** Operative data

Level of fusion	Estimated blood loss (mL)	Operative time (minutes)
All levels (n = 50)		
One-stage surgery (n = 13)	613 (150–1500)	351 (176–510)
Two-stage surgery (n = 37)	763 (25–2500)	482 (83–546)
Stage 1	327 (25–2100*)	192 (83–531)
Stage 2	463 (100–2500*)	291 (153–546)
Upper instrumented levels		
Lumbar (L1-L2) (n = 12)	570 (200–1500)	398 (267–520)
Lower thoracic (T10–T12) (n = 31)	754 (150–2400*)	479 (156–959)
Upper thoracic (T3–T5) (n = 7)	952 (300–2100^†^)	425 (217–561)

Values are expressed as mean, with range in parentheses; * one patient with an estimated blood loss of 2400 mL was an extreme outlier who had a retrocapsular renal hematoma; ^†^the one patient with an estimated blood loss of 2100 mL had severe osteoporosis.

The mean VAS and ODI scores decreased from 5.7 and 44 preoperatively to 2.9 and 22 at last followup (p < 0.001 and 0.03, respectively) (Table 3). The mean SF-36 score increased from 48 preoperatively to 74 at last followup (p = 0.026).

**Table 3:** Clinical and functional outcomes

Time of assessment	Mean score (points)		
	VAS pain	ODI	SF-36
Preoperative	5.7	44	48
6 months	2.2	32	55
p value*	< 0.001	0.011	0.029
12 months	2.5	27	64
p value*	< 0.001	0.004	0.007
24 months	2.4	24	70
p value*	< 0.001	< 0.001	0.007
36 months	2.7	27	73
p value*	< 0.001	0.015	0.005
> 36 months	2.9	22	74
p value*	< 0.001	0.03	0.026

* Compared with preoperative scores; ODI = Oswestry Disability Index.

The mean Cobb decreased from 42° (range, 30°–75°) preoperatively to 16° (range, 4°–46°) postoperatively (p < 0.001) (Table 4). The mean sagittal vertical axis decreased from 51 mm (range, 12–137 mm) to 27 mm (range, 0–84 mm) (p < 0.001). The mean coronal balance decreased from 30 mm (range, 4–143 mm) to 14 mm (range, 0–42 mm) (p < 0.001). The mean lumbar apical vertebral translation decreased from 41 mm (range, 11–88 mm) to 17 mm (range, 3–41 mm) (p < 0.001). The mean pelvic incidence-lumbar lordosis mismatch decreased from 14° (range, 1°–33°) to 11° (range, 1°–27°). A total of 88% of patients (44 of 50) were confirmed to have achieved arthrodesis on CT scan.

**Table 4:** Radiographic outcomes for all patients

Variable	Preoperative	Long-term postoperative (latest followup)	p value
Cobb angle (°)	42 (30–75)	16 (4–46)	< 0.001
Sagittal balance (sagittal vertical axis) (mm)	51 (12–137)	27 (0–84)	< 0.001
Coronal balance (mm)	30 (4–143)	14 (0–42)	< 0.001
Lumbar apical vertebral translation (mm)	41 (11–88)	17 (3–41)	< 0.001
Pelvic incidence-lumbar lordosis mismatch (°)	14 (1–33)	11 (1–27)	< 0.001

Values are expressed as mean, with range in parentheses.

A total of 23 complications were noted in 21 patients (Table 5), resulting in an overall complication frequency of 42% (21 of 50 patients), including 10 delayed complications (of whom six patients had pseudarthrosis). The proportion of patients with perioperative complications was 22% (11 of the 50 patients). One patient developed an intraoperative renal capsular hematoma that was uneventful with no clinical sequelae. One patient had a ureteropelvic injury with DLIF and underwent nephrostomy and paracentesis. One patient developed an unrelated cerebellar hemorrhage that was satisfactorily evacuated with no residual effect. One patient had a foot drop after DLIF at L4-L5 and underwent a posterior decompression. Three patients developed a quadriceps palsy, of whom two recovered in 6 months completely and one patient recovered to 4/5 motor strength by 18 months. Two patients had superficial sacral wound dehiscence and underwent débridement. There were three hardware issues revised with reinstrumentation and fusion. Two of these were symptomatic misplaced screws revised early and one was symptomatic prominent hardware revised late after fusion. There has been no breakage or failure of any of the screws or rods. There were six patients with pseudarthrosis all at L5-S1 and all had axial lumbar interbody fusion. There was loosening of the axial lumbar interbody fusion screw and or loosening of the sacral screws with increasing clinical pain. These were revised with either revision posterior instrumentation and extension to the pelvis or removal of the screw and then anterior lumbar interbody fusion with extension to the pelvis posteriorly. Three patients needed late secondary decompression, one for heterotopic ossification and two for persistent stenosis. Two patients developed late adjacent segment degeneration and proximal junctional kyphosis.

**Table 5:** Complications including need for revision surgery

Complication	Number of patients	Intervention
Superficial wound dehiscence	2	Local wound care
Pseudarthrosis	6	Revision AP fusion and/or extension to pelvis
Radiculopathy, stenosis	2	Microdecompression
Radiculopathy, heterotopic ossification	1	Laminoforaminotomy
Misplaced hardware	3	Reinstrumentation and fusion
Proximal junction kyphosis	2	Posterior instrumentation and fusion
Quadriceps palsy	3	2 recovered in 6 months, 1 at 18 months
Foot drop after direct lateral interbody fusion	1	Posterior decompression
Idiopathic cerebellar hemorrhage	1	Suboccipital craniectomy and hematoma evacuation, ventriculostomy placement
Renal capsular hematoma	1	CT angiography and observation
Ureteropelvic junction injury	1	Nephrostomy and paracentesis
Total complications	23	
Total patients with complications	21	

## Discussion

In the surgical treatment of AIS, the surgical goal of scoliosis surgery is achieving spinal balance in the sagittal and coronal planes [9]. Scoliosis curves tend to be stiffer in adults than in adolescents; as a result, release techniques such as facet resections or osteotomies are often called for before curve correction. Posterior column osteotomies allow for increased mobilization of the spine and correction in both the sagittal and coronal planes, but surgical time and blood loss increase with performance of osteotomies, and such operative intervention may be considered hazardous in elderly patients, given their increased risk for cardiovascular morbidity [1, 5, 8]. It is therefore reasonable to look for approaches to adult scoliosis that involve less blood loss and lower overall morbidity. cMIS fusion has been associated with decreased blood loss, decreased hospital stays, and reduced pain medication requirements when compared with open techniques [17, 20, 23], but to our knowledge, the utility of a cMIS approach specifically for AIS has not been specifically studied. We therefore evaluated these procedures in terms of surgical time and blood loss, patient-reported outcomes, deformity correction and fusion rate, and frequency and types of complications.

This study had a number of limitations. First, the study was retrospective. A larger study with a control group would have obvious advantages over this. Additionally, curves of greater than 75° were not treated this way. Thus, this study does not answer the role of the cMIS in more severe AIS. One other limitation is the careful selection of our patients as we gained experience with the techniques and this certainly could be seen as a selection bias. We chose only flexible or stiff curves to be treated in this manner and excluded any patient who had a rigid curve as evidenced by fused segments on preoperative CT scans. We excluded patients with any prior retroperitoneal surgery, osteoporosis with a T-score of less than −2.0, significant medical comorbidities that would preclude major spinal reconstruction, and debilitated deconditioned ambulatory status. Hence, our selective indications for this technique may tend to inflate the apparent benefit and safety of such treatment.

Our blood loss results seem more favorable compared to those of open series. Seo et al. [21], reporting outcomes in 152 patients older than 20 years undergoing open adult scoliosis correction, noted a mean blood loss of 2855.8 ±1822.9 mL. Guay et al. [15] in their study looking at risk factors for blood loss in surgery for idiopathic scoliosis noted a mean blood loss of 1971 ± 831 mL. The authors noted a correlation between the number of levels fused and duration of surgery with bleeding. Yu et al. [27] also noted that a number of fused levels of more than six, a preoperative Cobb angle of 50° or more, and osteotomy were risk factors for massive hemorrhage in scoliosis correction. In contrast, our total blood loss for our cMIS procedures averaged 613 mL when performed in a single setting or 763 mL when staged. Further, none of our patients needed to go to the intensive care unit and we believe this is certainly favorable for their postoperative course.

We noted improvements in functional clinical outcomes in terms of VAS, ODI, and SF-36 using this technique. The ODI improvement was similar to that reported by Yadla et al. [26] in a systematic review of open adult scoliosis correction (mean postoperative reduction in ODI of 15.7 for 911 patients).

Mean curve reduction in our series was by 63% or 26°. This is comparable to the mean correction noted by Yadla et al. [26] (40.7% or 26.6°). Of note, our mean preoperative sagittal vertical axis was 51 mm, which improved to 27 mm at last followup. Given that sagittal balance is a key determinant of patient clinical outcomes after undergoing spinal deformity correction [13, 14], we would caution against patients undergoing cMIS scoliosis correction techniques when the sagittal vertical axis is greater than 100 mm. This has been reported elsewhere [6, 7]. Additionally, careful attention should be paid to pelvic parameters in the surgical decision-making process for scoliosis and deformity correction. It is important to understand the limitations and ceiling effects of cMIS correction in the treatment of adult scoliosis. Given the limitations noted, in patients with significant sagittal imbalance (sagittal vertical axis > 10 cm and/or pelvic incidence-lumbar lordosis mismatch > 40°), adjunct techniques such as anterior longitudinal ligament release with lateral fusion [11] and/or posterior column osteotomies should be considered. Six of our 50 patients (12%) developed a pseudarthrosis, which was similar to the 12.9% pseudarthrosis rate noted by Yadla et al. [26] in their systematic review. All pseudoarthroses in our patients were at L5-S1 and all were in patients who underwent an initial axial lumbar interbody fusion. All six patients had sagittal imbalance preoperatively and this has resulted in us changing our protocol to using axial lumbar interbody fusion at L5-S1 only in patients who have preexisting acceptable sagittal parameters.

The proportion of our patients who had early and late complications (including pseudarthrosis and late adjacent segment degeneration) was comparable, if not favorable, when compared to that of open series. Cho et al. [10] found that 45.2% of their patients had early complications after primary spinal fusion for either AIS or de novo (degenerative) scoliosis; in the study of Kasliwal et al. [18], the proportion was 43% among patients undergoing primary adult scoliosis correction for either AIS or de novo scoliosis. Only two of the 36 patients in our series with radicular pain had persistent leg pain after surgery sufficient to undergo a secondary decompression. Thirty-four of the 36 patients developed relief of their leg pain by indirect decompression afforded through the lateral transpsoas fusion technique. The two patients who failed indirect decompression both had significant central canal stenosis.

cMIS percutaneous long-segment fusion represents a newer method of achieving surgical correction in patients with AIS. Our study shows that patients with AIS undergoing cMIS scoliosis correction have favorable radiographic improvement and good functional outcomes. This approach may be useful for moderate AIS without significant sagittal imbalance (sagittal vertical axis > 10 cm) or truly rigid fused curves. Patients had an overall low morbidity and complication rate at both early and late followup. cMIS strategies may obviate the need for routine facet resections and osteotomies in selected cases of AIS where curves are not truly rigid and significant sagittal correction is not desired.
